# Hemolysis-Inspired, Highly Sensitive, Label-Free IgM Detection Using Erythrocyte Membrane-Functionalized Nanomechanical Resonators

**DOI:** 10.3390/ma15217738

**Published:** 2022-11-03

**Authors:** Taeha Lee, Woong Kim, Jinsung Park, Gyudo Lee

**Affiliations:** 1 Department of Biotechnology and Bioinformatics, Korea University, Sejong 30019, Korea; 2 Interdisciplinary Graduate Program for Artificial Intelligence Smart Convergence Technology, Korea University, Sejong 30019, Korea; 3 Department of Mechanical Engineering, Hanyang University, Seoul 04763, Korea; 4 Department of Biomechatronics Engineering, Sungkyunkwan University, Suwon 16419, Korea

**Keywords:** immunoglobulin M, erythrocyte, cell membrane, microcantilever, resonant frequency

## Abstract

Immunoglobulin detection is important for immunoassays, such as diagnosing infectious diseases, evaluating immune status, and determining neutralizing antibody concentrations. However, since most immunoassays rely on labeling methods, there are limitations on determining the limit of detection (LOD) of biosensors. In addition, although the antigen must be immobilized via complex chemical treatment, it is difficult to precisely control the immobilization concentration. This reduces the reproducibility of the biosensor. In this study, we propose a label-free method for antibody detection using microcantilever-based nanomechanical resonators functionalized with erythrocyte membrane (EM). This label-free method focuses on the phenomenon of antibody binding to oligosaccharides (blood type antigen) on the surface of the erythrocyte. We established a method for extracting the EM from erythrocytes and fabricated an EM-functionalized microcantilever (MC), termed EMMC, by surface-coating EM layers on the MC. When the EMMC was treated with immunoglobulin M (IgM), the bioassay was successfully performed in the linear range from 2.2 pM to 22 nM, and the LOD was 2.0 pM. The EMMC also exhibited excellent selectivity compared to other biomolecules such as serum albumin, γ-globulin, and IgM with different paratopes. These results demonstrate that EMMC-based nanotechnology may be utilized in criminal investigations to identify blood types with minimal amounts of blood or to evaluate individual immunity through virus-neutralizing antibody detection.

## 1. Introduction

Immunoglobulin (Ig), also known as an antibody, is a glycoprotein that binds to an antigenic epitope [1]. Ig plays an essential role in the immune response by recognizing and binding to viruses and inducing elimination [2]. Ig takes different forms depending on the isotype, each with a different target specificity and role [3,4,5,6,7]. In particular, IgM is related to innate immunity, and the detection of IgM is important for immunoassays, such as determining the degree of neutralization of antibody formation [8,9,10]. So far, IgM has been mainly used as a molecular probe for the labeling method because it is capable of an antigen–antibody reaction, regardless of molecular orientation, in the form of five IgG bounds [11,12]. An example would be the use of IgM for the ABO blood type test [13].

Immunoassay is based on a biological method that analyzes a specific reaction between an antigen and an antibody [14,15]. An immunoassay can be used to test antigens using antibodies, or vice versa, and is applied in various fields such as disease diagnosis [16], drug screening [17], and healthcare [18]. Representative immunoassay techniques include Western blot [19] and enzyme-linked immunosorbent assay (ELISA) [20]. Western blot is a method for detecting the presence of a particular antigen in a sample using antibodies, and ELISA is a method for examining the amount of specific antigens in a sample through an enzyme bound to the antibody. Western blot and ELISA methods are common methods for detecting antigens and antibodies. Still, both methods use complex detection procedures and either the primary or secondary enzyme must be labeled with fluorescent molecules [21]. However, these labeling methods cause false-positive/negative occurrences in biological assays due to alterations in physicochemical or intermolecular binding properties [22,23]. To solve this problem, label-free methods for biomolecular detection have been developed, such as electrochemical [24,25,26], surface plasmon resonance [27,28], and nanowell assay [29,30,31]. The label-free immunoassay methods are attracting attention because of their advantages, such as easy fabrication [32] and low cost [33], while excluding the effect of the labels [34].

MC-based sensors have been widely used in immunoassays due to their advantages of high sensitivity [35], low cost [36], simple procedure [37], and low analyte requirements [38] for viruses [39], bacteria [40,41,42], antigens [43], and proteins [44]. The resonant frequency (*ω*) of the MC is inversely proportional to the square root of the mass of the MC. That is, an increase in the mass of the MC due to molecular adsorption decreases the *ω* of the MC. Using the *ω* shift (Δ*ω*), defined as *ω* − *ω*_0_, it is possible to assay biomolecules such as viruses [39], bacteria [40,41,42], antigens [43], and proteins [44]. Furthermore, recent studies have demonstrated that the MC-based detection of very low biomolecular concentrations, from nanograms to attograms per mL, is possible [45,46,47,48,49]. Despite these strong points, an MC-based IgM assay has never been attempted before due to the lack of technology. Meanwhile, we noted that IgM interacts with oligosaccharide antigens on erythrocytes during hemolysis, a symptom of autoimmune disease [50,51]. Specifically, when exogenous erythrocytes enter the body, IgM binds and removes them through hemolysis. In autoimmune diseases, IgM induces hemolysis by binding to endogenous erythrocytes. In detail, as the amount of bound IgM increases, the mechanical stress applied to the cell membrane increases, resulting in hemolysis [52].

**Figure 1:**
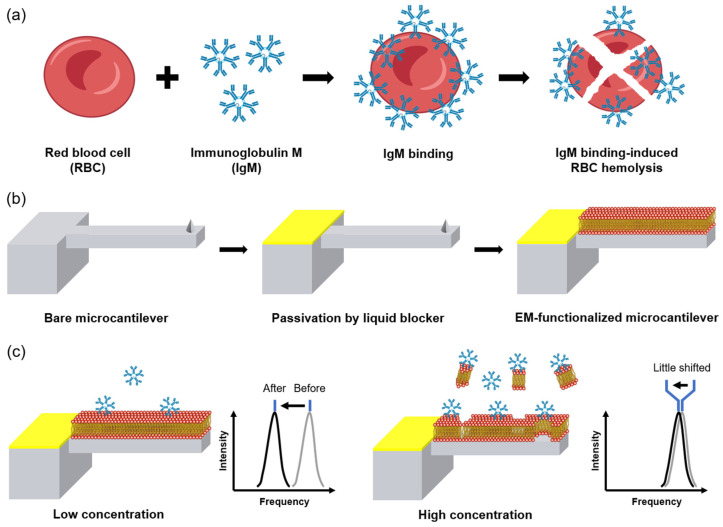
Schematic illustration of (**a**) hemolysis of erythrocyte due to high concentration of IgM and (**b**) the EMMC fabrication process. The base and MC are shown in gray, the liquid blocker in yellow, and the EM in red. (**c**) EMMC illustration and Δ*ω* at low and high concentrations of IgM.

Herein, inspired by IgM-induced hemolysis, we have fabricated a novel EM-functionalized MC (EMMC) for IgM detection (Figure 1a,b). EM was extracted from whole human blood and functionalized with a single layer on an MC. The surface functionalization of EM on the MC has two advantages over conventional receptor functionalization on the MC [53,54,55,56,57,58,59,60]. First, the cumbersome process of receptor immobilization can be omitted. Additionally, no additional molecular passivation is required between the receptors [39]. Recently, the EM functionalization of nanoparticles [53,54], electrodes [55,56,57,58,59], and paper sensors [60] has conferred highly sensitive and selective ligand–receptor binding, as well as the sophisticated filtration of interfering molecules. In this study, we demonstrated the detection performance of an EMMC as a function of IgM concentration (Figure 1c). We also conducted a selectivity test in the presence of interfering molecules such as human serum albumin, γ-globulin, and nonspecific IgM with different paratopes. We believe that the EMMC will be a robust and label-free method used to detect IgM that can be applied in various fields such as immune disorder diagnosis, immune status assessment, and neutralizing antibody quantification.

## 2. Materials and Methods

### 2.1. Chemicals

Advanced PAP pen (liquid blocker, 3-Bromopropane 65%, Ligroine 10%), distilled water (DW), deionized water (DIW), phosphate buffer saline (1×, PBS, pH 7.4), human serum, human serum albumin (HSA), and γ-globulin were purchased from Sigma Aldrich (Burlington, MA, USA). Additionally, 18:1 PE CF fluorescent lipid was purchased from Avanti Polar Lipid (Alabaster, AL, USA). Anti-A (ABO1) and Anti-B (ABO2) were purchased from DIAGAST (Loos, Nord, France).

### 2.2. Apparatus and Measurements

Morphology and fluorescence images of the MCs were characterized by microscopy (DS-Ni2, Nikon, Melville, NY, USA). An MC (PR-T190, *k* = 45 N/m; Probes, Seoul, Korea) was mounted on to an AFM aqua head (ezAFM, NanoMagnetics Instruments, Oxford, UK). The laser voltage before MC tuning was about 2.0V. Next, the *ω* of the MC was calibrated using the ‘Auto Tune’ method in the ezAFM v10.20 software. All the MCs were mounted on to AFM aqua heads after they were completely dried.

### 2.3. EM Collection

EM was extracted from human whole blood using the previously mentioned method [60]. The blood was extracted from a healthy 25-year-old male. Extracted blood was stored in an EDTA vacutainer and then immediately stored in the refrigerator. To extract the erythrocytes from the blood, the blood was centrifuged at 200× *g* for 5 min and washed three times with 1× PBS. Next, to lyse the erythrocytes, they were diluted 1:3 in a cold hypotonic solution (0.25× PBS) and stored in the refrigerator for 1 h. Then, to obtain pure EM, they were centrifuged under specific conditions (10,000× *g*, 1 h, and 4 °C) and washed 3 times with 1× PBS. The obtained clear ruby-colored EM was stored in a freezer (−80 °C). Before use, each EM solution was diluted with DW, and then sonicated and vortexed for 5 min.

### 2.4. EM Functionalization

Several steps were taken to functionalize the EM in the device. First, a liquid blocker was coated only on the base of the AFM probe (PR-T190) and dried at 23 °C for 5 min. Coating the base with a liquid blocker allows the EM to selectively functionalize only on the MC surface. Specifically, the MCs were functionalized by slowly pipetting 60 μL of EM solution (1%) and washing them 100 times to resolve EM residues.

### 2.5. Theory

The MC’s resonance frequency (*ω*) in dry air follows the elastic continuum model [61].

(1) $$\omega ={\left(\frac{\beta }{L_{MC}}\right)}^{2}\sqrt{\frac{EI}{\rho_{MC}A}}\equiv \sqrt{\frac{K_{MC}}{M_{MC}}}$$

where *L_MC_*, *EI*, *ρ_MC_*, *A*, *K_MC_*, and *M_MC_* are the MC’s length, bending rigidity, density, cross-sectional area, the MC’s effective stiffness, and the MC’s effective mass, respectively. In detail, *K_MC_* = *β*^4^*EI*/*L_MC_*^3^ and *M_MC_* = *ρ_MC_A.* The *β* satisfies the transcendental equation such that cos*β*cosh*β* + 1 = 0. Here, the dimensions of the MC are given by *L_MC_* × *W_MC_* × *T_MC_* (length × width × thickness), where *L_MC_* = 215 μm, *W_MC_* = 37.5 μm, and *T_MC_* = 8 μm. Following this equation, our MC’s *ω* is 149.54 kHz. The average of *ω* was experimentally obtained to be 153.25 ± 1.58 kHz. This shows that our MC satisfies the elastic continuum model. Because the *T_MC_* of the MC is much larger than that of the molecular layer, the mass is the main factor causing the resonant frequency shift (Δ*ω*) [62]. Therefore, in our experiments, the IgM molecule is responsible for increasing the mass. This can be expressed in terms of the Δ*ω* in the air as follows:

(2) $$\Delta \omega =\omega -\omega_{0}\propto \Delta M\equiv \left(M_{MC}-M_{MC0}\right)$$

where Δ*ω* is the resonant frequency shift measured in air, and Δ*M* is the total mass of the molecule, including molecular interactions. The EMMC can detect IgM using this Δ*ω*.

### 2.6. Measurement of Immunoglobulin by EMMC

For the IgM detection, various concentrations of IgM solution (60 μL) were dispensed on to the EMMC. Afterward, the EMMC was shaken at 60 rpm for two hours at 23 °C. The IgM-bound EMMC was carefully washed with PBS and fully dried. The EMMC’s *ω* was measured through frequency sweeping (see Section 2.2).

## 3. Results and Discussion

### 3.1. EMMC Fabrication

**Figure 2:**
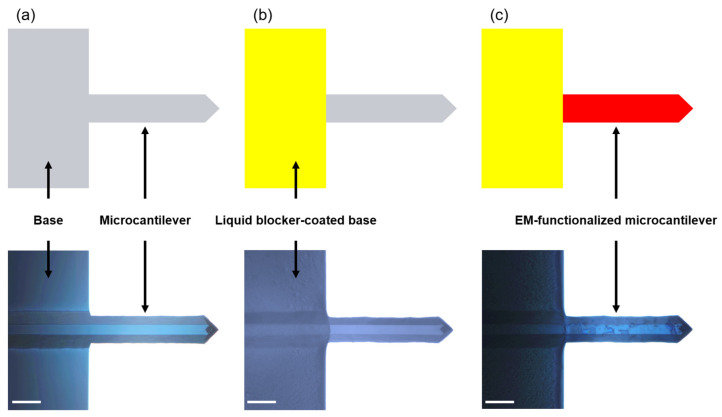
Schematic illustration and microscopic images (top view) of (**a**) bare MC, (**b**) bare MC with liquid-blocker-coated base, and (**c**) EM-functionalized microcantilever (EMMC). Liquid-blocker-coated base and EMMC are shown in yellow and red, respectively. Scale bar, 50 μm.

During the EM functionalization process, it is difficult to coat only the MC surface and not the MC base. To solve this problem, scientists use microinjection or microcapillary technology, which is considered to be a major limitation in the mass production of EMMCs [63,64]. To prevent the unwanted coating of EM on the base surface, we coated the surface of the base with a liquid blocker, which is a hydrophobic matter (Figure 2a,b). Basically, EM functionalization is only possible on the MC surface due to the liquid barrier coated on the base part. To confirm whether the liquid blocker affects the *ω* of the MC, we compared the *ω* of the MC before and after the liquid blocker coating was applied. The *ω* of the MC with the liquid-blocker-coated base was slightly different from that of the bare MC. Subsequently, EM was functionalized on the MC. In order to fabricate the EMMC, it is important to coat only a single layer of the EM on the MC surface. The two variables of EM concentration and the number of washes are very important. If high EM concentration was treated on the bare MC, the number of washes of the EMMC should be increased to remove excessive EM residues. These variables will depend on the type of materials and the shape of the MC. We can only describe the conditions for fabricating an EMMC based on the MC (PR-T190, Probes, Korea): agitate 100 times by repetitively pipetting with 1% EM solution (60 μL), and subsequently wash 100 times. As shown in Figure 2c, the EMMC shows distinctly functionalized EM compared to the bare MC.

**Figure 3:**
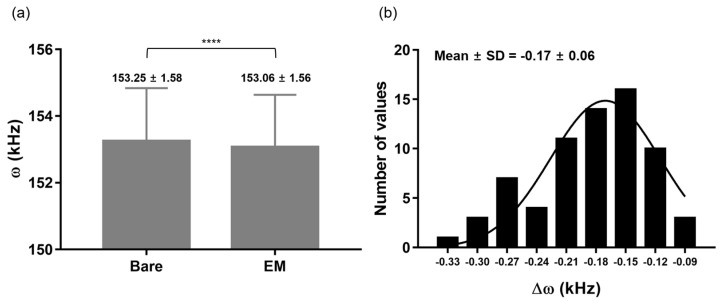
(**a**) *ω* before and after EM coating on the MC surface. (**b**) Histogram of Δ*ω* between bare MCs and EMMCs. **** *p* < 0.0001; (*n* = 66) Abbreviation note: erythrocyte membrane (EM), and standard deviation (SD).

To investigate how much Δ*ω* occurs due to EM coating, the MC’s *ω* was measured before and after the EM coating was applied. The *ω* for the bare MCs and the EMMCs are 153.25 ± 1.58 and 153.06 ± 1.56 kHz (mean ± standard deviation); a 0.17 ± 0.06 kHz decrease was observed after EM functionalization on the MC surface (Figure 3a,b). Based on Equation (2) above, a 0.17 ± 0.06 kHz shift indicates that the weight of the EM coated on the MC was about 35.5 pg. Additionally, the Gaussian distribution of the histogram for Δ*ω* (Figure 3b) implies that the EM was uniformly functionalized during the EMMC fabrication.

### 3.2. Immunoglobulin Detection and Selectivity Test Using EMMC Sensor

**Figure 4:**
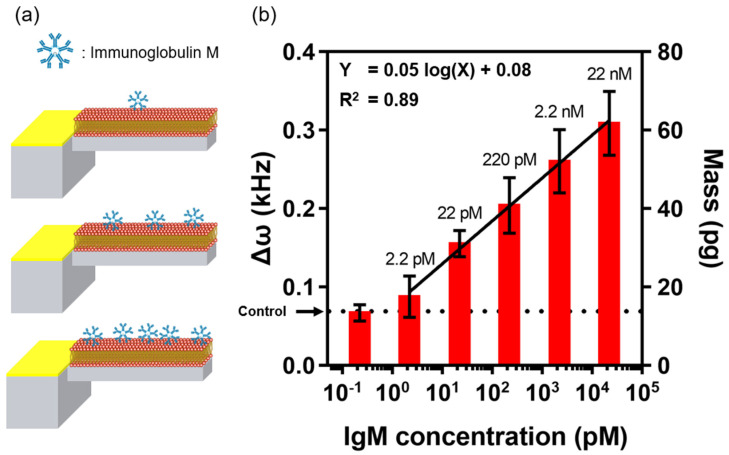
(**a**) Schematic illustration of IgM assay using the EMMC. (**b**) Δ*ω* and mass of EMMC as a function of IgM concentration. The dotted line indicates the value of Δ*ω* and mass of the control experiment. For each IgM concentration, a triplicate measurement was performed.

To check the performance of the EMMC in IgM detection, we treated it with various IgM concentrations (2.2 pM–22 nM) dissolved in 1× PBS (pH 7.4). Here, Δ*ω* is the difference in *ω* between the IgM-bound EMMC and the bare EMMC. As shown in Figure 4, the Δ*ω* values were 0.09, 0.16, 0.20, 0.26, and 0.31 kHz, as a function of IgM concentration (2.2 pM–22 nM). This suggests that IgM binds proportionally to antigens present on the EM surface. This plot also shows that Δ*ω* and log(IgM) are linearly related. The analyzed semi-log regression analysis was ‘Δ*ω* = 0.05 × log(X) + 0.08′, and the limit of detection (LOD) calculated as 3.3 × (σ/S) was 2.04 pM. The σ is the standard deviation of the response, and S is the slope of the linear fit. According to Equation (2), 2.04 pM LOD corresponds to 17.32 pg IgM, which can be considered the resolution of the EMMC in IgM detection (Table S1).

**Figure 5:**
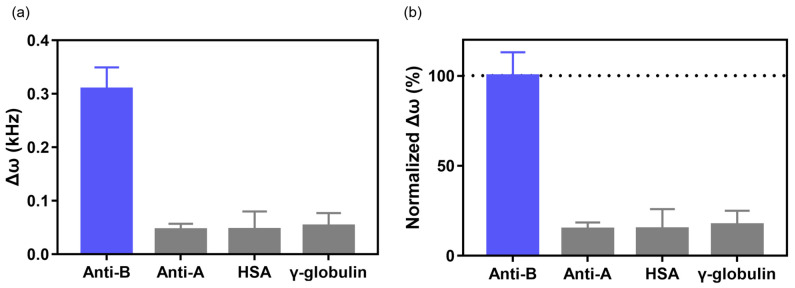
Selectivity of the EMMC. (**a**) Δ*ω* and (**b**) normalized Δ*ω* of the EMMC with interfering proteins. The selectivity test of EMMC with nonspecific IgM (Anti-A) and other interfering proteins (i.e., γ-Globulin and human serum albumin (HSA)). The IgM (Anti-B) and IgM (Anti-A) level was 22 nM, and the concentrations of all other interfering proteins were the same (0.1 mg/mL).

To check the selectivity of our EMMC, we considered different types of antigens. A human blood type is determined by the type of antigen present on the surface of the erythrocytes. Blood type A contains antigens only (Type A), whereas blood type B contains antigens (Type B) only on erythrocytes. Therefore, type B erythrocytes do not interact with IgM (Anti-A) and bind selectively to IgM (Anti-B). Note that our EMMC was fabricated with erythrocytes from blood type B. The selectivity of the EMMC sensor was verified by measuring high concentrations of major proteins present in the blood, such as IgM (Anti-A), HSA, and γ-globulin [59]. The treated IgM (Anti-A) concentration was 22 nM, and the amounts of HSA and γ-globulin were 0.1 mg/mL. The Δ*ω* values of the EMMC treated with IgM (Anti-A), HSA, and γ-globulin were 0.05 ± 0.01, 0.05 ± 0.03, and 0.05 ± 0.02 kHz, respectively (Figure 5a). The intensities of Δ*ω* for IgM (Anti-A), HSA, and γ-globulin normalized by Δ*ω* values in the IgM (Anti-B) condition were 14.79 ± 3.05, 14.90 ± 9.04, and 17.17 ± 6.41%, respectively (Figure 5b). These results suggest that the EMMC has high selectivity and distinguishes IgM (Anti-B) from other types of IgM and blood proteins.

### 3.3. EM Exfoliation at High IgM Concentration

**Figure 6:**
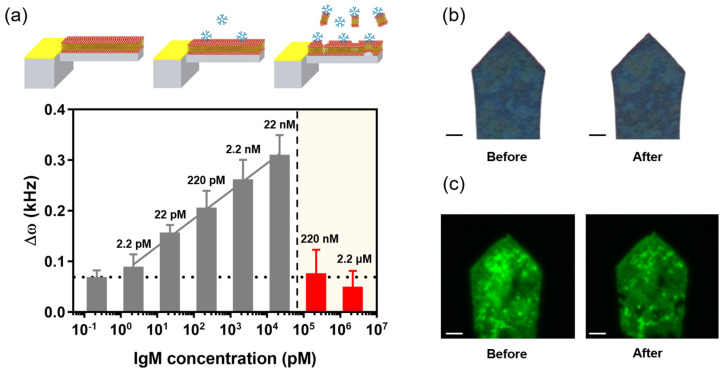
(**a**) Δ*ω* according to the low (2.2 pM–22 nM, gray) and high IgM concentration (220 nM–22 μM, red). (**b**) Microscopic and (**c**) fluorescence images before and after high-concentration IgM (220 nM) binding with the EMMC. For each IgM concentration, a triplicate measurement was performed. Scale bar, 10 μm.

We investigated the detection ability of the EMMC at high IgM concentrations. Interestingly, the *ω* of the EMMC decays sharply at high concentrations of IgM (>220 nM) and remains almost unchanged. Specifically, the Δ*ω* of EMMCs exposed to 220 nM and 22 μM IgM were 0.07 ± 0.04 and 0.05 ± 0.03. This implies that the EMMC will normally operate below 22 nM IgM, but may malfunction above 220 nM (Figure 6a). This happens when EM detachment occurs due to large amounts of IgM being bound to the EM, similar to hemolysis by IgM. To confirm this, the surface of the EMMC was observed through an optical microscope. While comparing the EMMC surfaces before and after high concentrations of IgM were introduced, and we found that some of the EM peeled off from the MC surface (Figure 6b). Moreover, these differences were clearly visible in the fluorescence micrographs (Figure 6c). From these results, it is reasonable to say that high concentrations of IgM can partially remove the EM from the EMMC. This may be due to the structure of IgM, a pentamer characterized by 10 binding sites. As the amount of IgM bound to the EMMC increases, the mechanical stress applied to the EM increases, which eventually leads to the delamination of the EM. This exfoliation effect may help better understand the mechanical properties of cell membranes, including EM. Cell membrane-functionalized MCs, which enable the study of the destruction mechanism of the cell membranes by bound molecules, can also be taken as an example, which may aid a better understanding of various necrosis-induced diseases. In addition, studies are needed to stably coat EMs on MC surfaces via various methods other than physical adsorption (*e.g.,* covalent bonding). This is indeed challenging and would be a desirable research theme for future work.

### 3.4. IgM Detection in Human Serum

**Figure 7:**
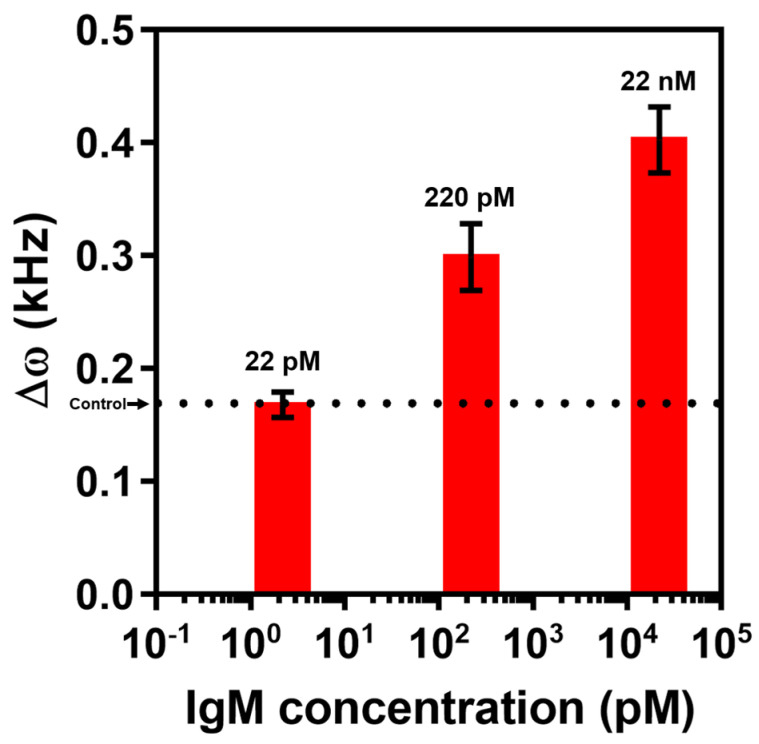
Δ*ω* of EMMC as a function of IgM concentration in human serum. The dotted line indicates the value of Δ*ω* for the control experiment (i.e., serum only). For each IgM concentration, a triplicate measurement was performed; they are plotted as mean ± standard deviation.

To investigate the practicality of EMMC, the IgM assay based on human serum was performed using the EMMC. Type B IgM was spiked into the human serum at concentrations around 0–22 nM, which was identical to the range used in the bioassay that was completed with PBS (Figure 4). IgM dissolved in the human serum was detected by the EMMC, and the result showed a linear relationship between Δ*ω* and IgM concentration (0–22 nM) under a semi-logarithmic scale (Figure 7). This result implied that high-sensitivity IgM detection is doable using the EMMC even in the use of real sample which contains a lot of interfering molecules such as albumin, glucose, urea, creatinine, and so on. By utilizing the EMMC for IgM detection, it will be able to diagnose the individual immune strength and to evaluate the vaccine efficiency based on neutralizing antibody level in various body fluids. For a further study, the EMMC will be capable of evaluating the designing and binding efficiency of synthetic antibodies, antiviral agents and disinfectants.

## 4. Conclusions

We have developed a nanomechanical resonator sensor called EMMC. The EM was uniformly and stably functionalized on the MC surface to achieve ultra-sensitive IgM detection. The linear detection ranges and LOD of the EMMCs for IgM detection were 2.2 pM–22 nM and 2.0 pM IgM. In addition, we confirmed that the EMMC has excellent selectivity through exposure to IgM (Anti-A), HSA, and γ-globulin. Our results prove that the EMMCs are highly sensitive and can selectively detect antibodies with a simple procedure and a label-free method. Although the EMMC successfully detected IgM in PBS and serum, further investigation is required for IgM detection in patient samples. We expect that our approach to EMMC fabrication and biosensing performance will shed light on the development of highly sensitive and selective label-free biosensors capable of detecting target molecules by single-step cell membrane functionalization.

## Data Availability

Data available on request due to restrictions, e.g., privacy or ethical.

## Supplementary Materials

The following supporting information can be downloaded at: https://www.mdpi.com/article/10.3390/ma15217738/s1, Table S1. Comparison of various methods for IgM detection with our work. References [65,66,67,68,69] are cited in the Supplementary Materials.
