# Supplementary material for: Hemolysis-Inspired, Highly Sensitive, Label-Free IgM Detection Using Erythrocyte Membrane-Functionalized Nanomechanical Resonators
Source: Materials (Basel). 2022 Nov 3;15(21):7738. doi: 10.3390/ma15217738 (PMC9654754; doi:10.3390/ma15217738)
Supplement: Supplementary file 1 [file materials-15-07738-s001.zip › materials-1988948-supplementary.pdf]

Supplementary Information

Hemolysis-Inspired, Highly Sensitive, Label-Free IgM Detection Using Erythrocyte Membrane-Functionalized Nanomechanical Resonators

Taeha Lee [1,2,†], Woong Kim [3,†], Jinsung Park [4,*] and Gyudo Lee [1,2,*]

[1] Department of Biotechnology and Bioinformatics, Korea University, Sejong 30019, Korea
[2] Interdisciplinary Graduate Program for Artificial Intelligence Smart Convergence Technology, Korea University, Sejong 30019, Korea
[3] Department of Mechanical Engineering, Hanyang University, Seoul 04763, Korea
[4] Department of Biomechatronics Engineering, Sungkyunkwan University, Suwon 16419, Korea
* Correspondence: nanojspark@skku.edu (J.P.); lkd0807@korea.ac.kr (G.L.)
† These authors contributed equally to this work.

Table S1. Comparison of various methods for IgM detection with our work

Year	Sensor type	Molecular recognition type	Linear range	LOD	Selectivity test	Label-free method	Ref.
2018	Electrochemical	Sulfide quantum dots@polydopamine-labeled anti-IgM	0 – 0.5 mg/mL	130 ng/mL	No	No	1
2021	DNA-assisted nanopore sensing	N protein functionalized magnetic bead	0.05 – 10 μg/mL	50 ng/mL	No	No	2
2021	Surface Plasmon Resonance (SPR)	Anti-IgM	1.0 – 200 ng/mL	0.08 ng/mL	Yes	Yes	3
2022	Electrochemical	Molecularly imprinted polymers	1 pg/mL – 100 ng/mL	0.58 pg/mL	Yes	Yes	4
2020	Lateral flow assay (LFA)	Selenium nanoparticle-modified N protein	20 – 50 ng/mL	20 ng/mL	No	No	5
2022	EMMC	Erythrocyte membrane with receptor molecule	2.2 pM – 22 nM (2.09 ng/mL – 20.9 μg/mL)	2.0 pM (1.9 ng/mL)	Yes	Yes	This work

Reference

1. G. A. Ortega, J. C. Zuaznabar-Gardona and E. Reguera, *Biosens. Bioelectron.*, 2018, **116**, 30-36.
2. Z. Zhang, X. Wang, X. Wei, S. W. Zheng, B. J. Lenhart, P. Xu, J. Li, J. Pan, H. Albrecht and C. Liu, *Biosens. Bioelectron.*, 2021, **181**, 113134.
3. N. Bereli, M. Bakhshpour, A. A. Topçu and A. Denizli, *Micromachines*, 2021, **12**, 1092.
4. Z. Liu, Z.-Z. Yin, G. Zheng, H. Zhang, M. Zhou, S. Li and Y. Kong, *Bioelectrochemistry*, 2022, **148**, 108267.
5. Z. Wang, Z. Zheng, H. Hu, Q. Zhou, W. Liu, X. Li, Z. Liu, Y. Wang and Y. Ma, *Lab Chip*, 2020, **20**, 4255-4261.
